# Full immunization coverage and its associated factors among children aged 12–23 months in Ethiopia: further analysis from the 2016 Ethiopia demographic and health survey

**DOI:** 10.1186/s12889-019-7356-2

**Published:** 2019-07-30

**Authors:** Koku Sisay Tamirat, Malede Mequanent Sisay

**Affiliations:** Department of Epidemiology and Biostatistics, Institute of Public Health, College of Medicine and Health Sciences, University of Gondar, Gondar, Ethiopia

**Keywords:** Full immunization; 12–23 months children, Associated factors, Ethiopia

## Abstract

**Background:**

Vaccination is one of the cost effective strategies reducing childhood morbidity and mortality. Further improvement of immunization coverage would halt about 1.5 million additional deaths globally. Understanding the level of immunization among children is vital to design appropriate interventions. Therefore, this study aimed to assess full immunization coverage and its determinants among children aged 12–23 months in Ethiopia.

**Methods:**

The study was based on secondary data analysis from the 2016 Ethiopia Demographic and Health Survey (EDHS). Information about 1,909 babies aged 12–23 months was extracted from children dataset. Both bivariate and multivariable logistic regression models were utilized to assess the status and factors associated with full immunization. Adjusted odds ratio (AOR) with a 95% confidence interval (CI) was computed. Variables with less than 0.05 *p*-values in the multivariable logistic regression model were considered as statistically and significantly associated with the outcome variable.

**Results:**

The overall full immunization coverage was 38.3% (95% CI: 36.7, 41.2). Rural residence (AOR = 0.60, 95% CI: 0.43, 0.84), employed (AOR = 1.62, 95% CI: 1.31, 2.0), female household head (AOR = 0.58, 95% CI: 0.44, 0.76), wealth index [middle (AOR = 1.44, 95% CI: 1.07, 1.94) and richness (AOR = 1.65, 95% CI: 1.25,2.19)], primary school maternal education (AOR = 1.38,95% CI: 1.07, 1.78), secondary school maternal education (AOR = 2.19, 95% CI: 1.43, 3.36), diploma graduated mothers (AOR = 1.99, 95% CI: 1.09, 3.61), ANC follow ups (AOR = 2.79, 95% CI:2.17 3.59), and delivery at health facilities (AOR = 1.76, 95% CI: 1.36, 2.24) were significantly associated factors with full immunization.

**Conclusion:**

Full immunization coverage in Ethiopia was significantly lower than the global target. Female household head and rural dwellings were negatively associated with full immunization. In contrast higher maternal education, employment, middle and rich economic status, ANC follow up, and delivery at health facility were positively associated with full immunization among 12–23 months old children. This suggests that improved health education and service expansion to remote areas are necessary to step immunization access.

## Background

Vaccination is one of the prevention strategies for common childhood illnesses. It prevents morbidities and mortalities from diphtheria, hepatitis B, measles, mumps, pertussis, pneumonia, polio, rotavirus diarrhea, rubella, cervical cancer, and tetanus [1, 2]. Vaccine preventable diseases (VPDs) account for 17% of the global under five mortality per annum [3]. According to World Health Organization (WHO) 2017 report, 116.2 million infants (85%) received the third doses of DPT, and worldwide, 123 countries reached the third dose of diphtheria, pertussis, and tetanus (DPT3) coverage to 90%. Despite the increasing uptake of new and underused vaccines, still an estimated 19.9 million children under the age of 1 year have not received DTP3 vaccine [1, 4–6]. Further improvement of global immunization coverage would prevent an additional 1.5 million deaths [3]. According to a case-based surveillance, the annual incidence of measles was estimated at 29.1 cases per 1 million people [7].

Expanded program of immunization (EPI) was launched by WHO in 1974 with the objectives of reducing morbidity and mortality from six VPDs. Ethiopia started the EPI program in 1980 with a longer-term goal of achieving 90% DPT3 coverage in all regions through strategies of reaching every district (RED) and sustainable outreach service (SOS) approaches. In the Ethiopia health care system, immunization is provided free of charge and services are available from the smallest health post level to the highest hospitals [8].

According to guidelines developed by the World Health Organization (WHO), children are considered fully immunized when they have received one dose of Bacillus Calmette Guerin (BCG), three doses of DPT, three doses of polio vaccines, and one dose of measles vaccination by the age of 9–12 months [9, 10]. Ethiopia has incorporated Haemophilus influenza type B (HiB) and hepatitis-B (HepB) antigens to the previous DPT vaccines and replaced as Pentavalent vaccine (DPT plus Hep B and Hib) [1, 3, 8, 11]. A variety of vaccines, of which the Pneumococcal conjugate vaccine (PCV), Rota, and Human papilloma (HPV) vaccines were the most recent have been introduced into the national EPI service overtime. Different findings showed that the proportion of full immunization coverage in the country ranged from 36.6% in Somalia region to 100% in Addis Ababa [1, 4, 10, 12–17]. Factors associated with child full immunization included socio-demographic characteristics (maternal educational status and residence), health service delivery (place of delivery, ANC follow up, vaccine availability residence, and cold chain management) [1, 4, 10, 12–22].

Although some community based works are available with different findings, no study has shown the overall national full immunization coverage after the new vaccines have been introduced into the EPI schedule. Therefore, the objective of this study was to measure the full immunization coverage and associated factors among children aged 12–23 months in Ethiopia in order to help planners assess the progress of the national full immunization coverage.

## Methods

### Data source

The data used in this paper is from the 2016 Ethiopian Demographic and Health Survey report. Ethiopia is the second largest populous country in Africa with 102.4 million people and an annual population growth rate of 2.5%. The country is divided into nine regional and two-city administrations and has a three-tier health care system with the primary care facilities situated in nearby communities.

The two stage stratified sampling technique/ method was used for the survey. Initially, the enumeration area were stratified into urban and rural. The first stage involved selecting clusters, within the enumeration areas. The second stage was a systematic listing of households in the selected clusters. Out of each cluster 28 households were randomly selected to constitute the total sample size of households. Out of 7,193 women who gave birth in the past 5 years preceding of the survey, 5,980 were interviewed about the vaccination status of their children, and data gathered from 1,909 of the mothers who had children aged 12–23 months of were analyzed [23].

### Measurement of variables

Full immunization was the response variable, whereas socio-demographic characteristics (age, residence, religion, marital status), reproductive health history (place of delivery, birth order, antenatal care and postnatal care follow up) were the independent variables.

The information in the 2016 EDHS report on vaccination coverage was collected from immunization cards shown to the interviewers and from mothers’ verbal responses. When cards were available, the interviewer copied the vaccination dates directly onto questionnaires. When vaccination cards were not available for the child or if the vaccine was not recorded on the card as being given, the respondents were asked to recall if vaccine were given to her child.

According to the WHO guideline [1], “complete or full immunization” coverage is defined as a child that has received one dose of BCG, three doses of pentavalent, pneumococcal conjugate (PCV), oral polio vaccines (OPV); two doses of Rota virus and one dose of measles vaccine. We recoded each variable (vaccinations) as “0” and “1” for children who didn’t take the recommended doses and those who took, respectively, on the basis of the reports of mothers and information in the child vaccination card. Then we added all “0” and “1”s and labeled the total as “Immunization status”. The immunization status was recoded as “1” if the child had received all the recommended doses of all vaccinations and categorized as “full immunization” or “0” if the child had missed one or more doses of vaccinations and categorized as “Incomplete immunization”.

### Statistical analysis

Descriptive statistics were used to describe the level of full immunization coverage by socio-demographic characteristics. Bivariate and multivariable logistic regression analyses were conducted to identify the determinants of full immunization. Logistic regression was chosen because our dependent variable was dichotomous (i.e., 0 and 1). Variables in bivariable logistic regression analysis with *p*-values less than 0.2 were entered into the multivariable analysis. Adjusted odds ratio (AOR) and 95% confidence Interval (CI) were used to assess the strength of associations between the outcome and the independent variables. The threshold for statistical significance was set at *p* < 0.05. The whole analysis was performed using STATA version 15.0.

## Results

### Maternal and child socio-demographic characteristics

A total of 1,909 women with children aged 12–23 months were included in the final analysis. The majority (79.2%) women were rural dwellers and 61.1% of them had no formal education. The median age of the women was 28 (IQR: 24–33) years, and half of them were aged between 25 and 34 years; 47.3 and 31.5% were Muslims and Orthodox Christians, respectively. The poorest wealth quintile accounted for 34.3% of the participants the majority (93.5%) whom married, and about half (51%) of the children were male (Table 1).

**Table 1:** Socio-demographic characteristics of women with children 12–23 months of age in Ethiopia, 2016 (the relationship between Women’s autonomy 1909)

Characteristics	Category	Frequency	Percentage
Region	Tigray	218	11.42
	Afar	169	8.85
	Amhara	178	9.32
	Oromia	285	14.93
	Somali	219	11.47
	Benishangul Gumz	156	8.17
	SNNP	226	11.84
	Gambella	134	7.02
	Harari	117	7.02
	Adiss Abeba	99	5.19
	Dire Dawa	108	5.66
Residence	Urban	398	20.85
	Rural	1511	79.15
Age of women	15–24	539	28.23
	25–34	971	50.86
	35–49	399	20.9
Educational level	No formal education	1166	61.08
	Primary education	502	26.3
	Secondary education	156	8.17
	Diploma and higher degree	85	4.45
Sex of child	Male	936	49.03
	Female	973	50.97
Wealth quintile	Poor	975	51.07
	Middle	278	14.56
	Rich	656	34.36
Sex of house hold head	Male	1497	78.4
	Female	412	21.6
Marital status	Married	1787	93.5
	Other	122	6.5
Employment/occupation	Yes	795	41.6
	No	1114	58.4
Place of delivery	Health facility	1064	55.74
	Home	845	44.26
Religion	Orthodox	602	31.53
	Muslim	913	47.83
	Protestant	339	17.76
	Other	55	2.88
ANC follow up	Has ANC follow up	1227	64.27
	No ANC follow up	682	35.73
Birth order	1	409	21.42
	2–5	1022	53.5
	6+	478	25.04
Postnatal care	Yes	172	9.01
	No	1737	90.99
Parity	1	379	19.8
	2–5	1,046	54.8
	6+	484	25.4
Child size at birth	Small	549	28.8
	Average	769	40.3
	Large	591	30.9

### Immunization coverage in Ethiopia

**Fig. 1:**
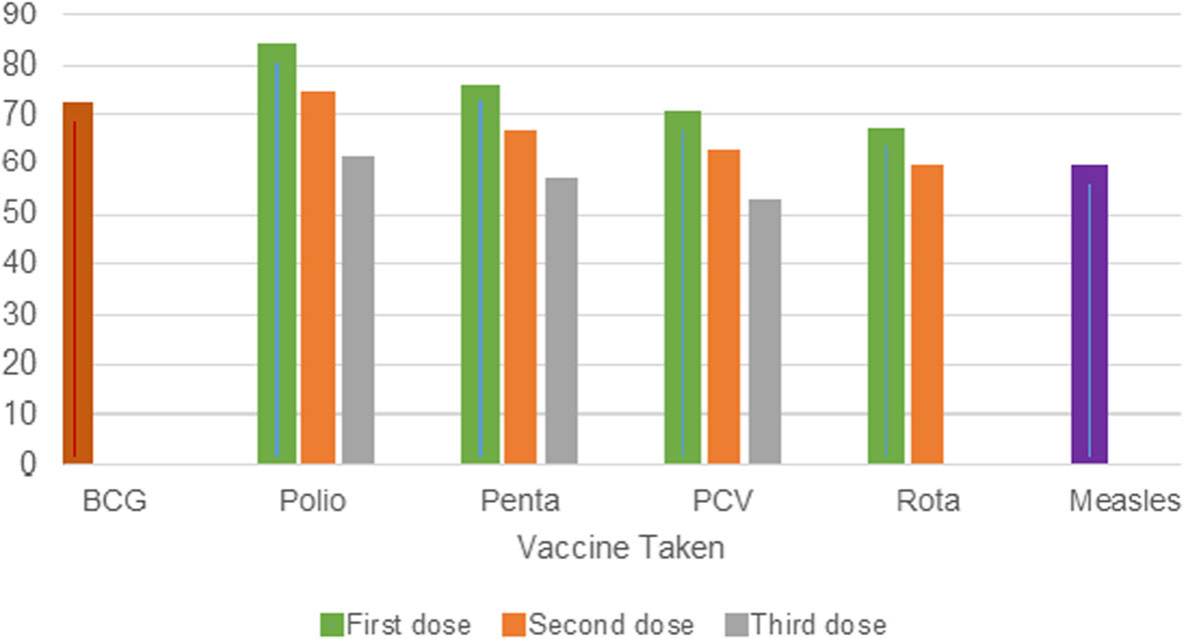
Vaccine specific immunization coverage among 12–23 month children in Ethiopia, 2016

In this study the overall full immunization coverage was 38.3% (95% CI: 36.7 41.2) according to the Ethiopian EPI schedule. Vaccine specific coverage for Pentavalent 3, OPV3, PCV3, Rota 2, and Measles were 56.1, 60.4, 51.9, 58, and 57.8%, respectively (Fig. 1). Full immunization coverage among rural dwellers was 31.7 and 66.6% in urban areas. Full immunization coverage was heterogeneous among Ethiopian administrative regions, ranging from 8.8% in Afar region to 86.8% in Addis Ababa (Table 2).

**Table 2:** Full immunization coverage among children aged 12–23 months in the Regional administrations of Ethiopia, 2016 (*n* = 744)

Regions	Frequency	Percentage
Tigray	138	63.3
Afar	15	8.8
Amhara	74	41.6
Oromia	73	25.6
Somali	44	20.1
Benishangul Gumz	82	52.6
SNNP	74	32.7
Gambella	36	26.8
Harari	56	42.7
Adiss Abeba	86	86.8
Dire Dawa	72	66.6

### Determinants of full immunization among children aged 12–23 months

In the bivariable logistic regression, maternal education, residence, household head, wealth, employment, sex of household head, ANC follow-up, and parity were significant at 0.2 *p*-value. In the multivariable logistic regression, only employment, residence, maternal education, wealth quintile, place of delivery, sex of household head, and ANC follow up were statistically significant at *p*-value of 0.05.

The odds of full immunization for rural women’s children decreased by 40% (AOR = 0.60, 95% CI: 0.43, 0.84) compared to those of urban dwellers. The odds of full immunization for the children of employed mothers were 1.62 (AOR = 1.62, 95% CI: 1.31, 2.0) times higher compared to those of unemployed mothers. The odds of full immunization of children whose mothers had primary (AOR = 1.38, 95% CI: 1.07, 1.78) and secondary (AOR = 2.19, 95% CI: 1.43, 3.36) school as well as diploma and above (AOR = 1.99, 95% CI: 1.09, 3.61) level of educational were higher than those of children whose mothers had no formal education. For women who had middle and rich wealth status the odds of full immunization of children were 1.44 (AOR = 1.44, 95% CI: 1.07, 1.94) and 1.65 (AOR = 1.65, 95% CI: 1.25, 2.19) times higher compared to those of poorer mothers. The odds of full immunization of children whose mothers had ANC follow ups during pregnancy were 2.79 (AOR = 2.79, 95% CI: 2.17, 3.59) higher than those of children whose mothers had no follow ups. For women who delivered in health facilities, the odds of full immunization of children were 1.76 (AOR = 1.76, 95% CI: 1.38, 2.24) times higher compared to those of children whose mothers delivered at home. The odds of full immunization of children whose household heads were female were 42% (AOR = 0.58, 95% CI: 0.44, 0.76) lower than those of their counterparts (Table 3).

**Table 3:** Bivariable and multivariable logistic regression analysis to identify factors associated with fully immunization among women with 12–23 month children in Ethiopia, 2016

Variables	Full Immunization		Crude odds ratio (OR 95% CI)	Adjusted odds ratio (OR 95% CI)
	Yes	No		
ANC follow up				
Yes	624	603	4.84(3.86 6.07)	2.79(2.17 3.59)*
No	120	562	1	1
Place of residence				
Urban	265	133	1	1
Rural	479	1032	0.23(0.18 0.29)	0.60(0.43 0.84)*
Place of delivery				
Health facility	480	365	3.98(3.28 4.84)	1.76(1.36 2.24)*
Home	264	800	1	1
Educational level				
No education	333	833	1	1
Primary	241	261	2.3(1.86 2.86)	1.38(1.07 1.78)*
Secondary	105	51	5.15(3.60 7.36)	2.19(1.43 3.36)*
Tertiary	65	20	8.13(4.84 13.36)	1.99(1.09 3.61)*
Household head				
Male	889	608	1	1
Female	276	136	0.72(0.57 0.90)	0.58(0.44 0.76)*
Wealth quintile				
Poor	248	727	1	1
Middle	110	168	1.91(1.45 2.54)	1.44(1.07,1.94)*
Rich	386	270	4.19(3.39 5.18)	1.65(1.25 2.19)*
Employment				
No	743	371	1	1
Yes	422	373	1.77(1.46 2.13)	1.62(1.31 2.0)*
Parity				
1	187	192	1	1
2–5	419	627	0.68 (0.54 0.86)	1.13(0.85 1.50)
6+	138	346	0.41(0.30 0.54)	0.99(0.70 1.41)

*shows a *p*-value less than 0.05

## Discussion

This study revealed that the overall full immunization coverage of Ethiopia was 38.3%, much lower than the 86% Government report and less promising to meet the 2020 health sector transformation plan of 95% [8, 23]. Vaccine specific full immunization coverage’s among children were 56.1% for Pentavalent third dose and 57.8% for Measles, below the Federal Ministry of Health 2015 report of 94% for both of them [8]. The possible reasons for the discrepancies between the national reports and this study might be spurious and false reports from health facilities.

This study also showed differences between full immunization and vaccine specific full dose coverage’s. The possible explanations for the variations might be the stock out of vaccines and the side effects of multiple injections. Furthermore as shown in the Table 2, full immunization coverage was in Ethiopia highly varies among administrative regions, ranging from 8.8% in Afar to 86.8% in Addis Ababa. The possible reasons might be socio-demographic and health seeking behavior differences among regions. In addition, most of the regions with low full immunization coverage had weak health care systems which led to low uptake of vaccines. Moreover, some of the regions like Afar and Somalia had hard to reach areas are nomadic and pastoralist inhabitants with no permanent residence.

This finding of full immunization coverage was lower than those of studies conducted in Togo (63.7%), Cameroon (53.6%), Timor-Leste (52.6%), Uganda (52.5%), Coted’Ivoire (50.5%) DR Congo (49.8%), and Haiti (45.8%) [1, 4, 20, 24, 25]. It was higher than findings in Somalia (11.6%), Mauritania (35.3), Nigeria (33.2%), Chad (11.4%), and the Republic of Central Africa (17.3%) [25, 26]. The possible explanations might be differences in study periods and number of vaccines Like PCV and Rota incorporated in the expanded program of immunization of Ethiopia. Health system differences among countries are also possible explanations for the observed differences. The full immunization coverage finding in this study was significantly higher than 2005 and 2011 EDHS reports of 19 and 24%, respectively [14]. This might be due to tremendous efforts of the Government to realize the millennium development goal of reducing child mortality from vaccine preventable diseases.

Maternal characteristics, residence, educational level, sex of household head, employment, wealth index, ANC follow up and place of delivery were factors associated with full immunization coverage among 12–23 months of age children. Rural residence was associated with lower full immunization of children compared to urban dwellings. This finding is in agreement with those of studies conducted in Arbaminch, Lay Armachio, and Jigijiga of Ethiopia, and Nigeria [9, 13, 14, 17]. This might be due to less accessibility of health facilities to EPI and differences in awareness about immunization. Maternal educational status of primary school and above associated with an increased full immunization of children. This finding is in agreement with the results of previous studies conducted in Ethiopia, Togo, Arbaminch, and Southwest Ethiopia [14, 17, 27–29]. The full immunization of children with female household heads was lower compared to children who had male household heads. This might be because of high workload and family responsibilities women may not heed EPI schedules for children vaccination. This finding was supported by that of a study conducted in Togo [20]. The full immunization of children whose mothers had ANC follow ups was three times higher than that of children whose mothers had no follow ups. This is supported by the results of previous studies conducted in Togo and Ethiopia [10, 12, 20, 30]. This might be because women who attend follow ANC may get counseling about child immunization in the postnatal period. The children of middle income and rich mothers were associated with higher full immunization than the children of poor mothers. This might be due to differences in child care practice, better health seeking behavior, and health care access. This finding was supported by studies conducted in Nigeria, Togo, and Southwest Ethiopia [9, 12, 14, 20, 22, 29]. The children of women who delivered at health facilities were two times more likely to receive full immunization compared to those of women who had home delivery. This finding was concordat with those of studies in Nigeria and Ethiopia [9, 17, 20, 22, 30]. This might be due to the fact that some vaccines, like BCG and OPV 0 are often given immediately after birth at health facilities. The children of employed mothers were associated with increased fully immunization compared to those of unemployed ones. This might be due to better information access about disease preventions, like immunization.

Sine not all children had vaccination cards, information about immunization status had to be limited the mothers’ verbal responses which were found to be prone to recall bias. Besides, having been based on secondary data analysis, this survey could not assess factors relating to the supply side and health system.

## Conclusion

Full immunization coverage in Ethiopia was significantly lower than the global target. Female household head and rural dwelling were negatively associated with full immunization. In contrast higher maternal education, employment, middle and rich economic status, ANC follow up, and delivery at health facility were positively associated with full immunization among 12–23 months old children. This suggests that improved health education and service expansion to remote areas are necessary to step immunization access.

## Data Availability

The datasets used during the current study is available from the corresponding author on reasonable request.

## Abbreviations

ANC: Antenatal care
AOR: Adjusted odds ratio
BCG: Bacilli Calamite Guerin
DPT: Diphtheria, pertussis, and tetanus
EA: Enumeration area
EDHS: Ethiopia Demography Health Survey
EPI: Expanded program of immunization
IQR: Interquartile range
OPV: Oral polio vaccine
OR: Odds ratio
PCV: Pneumococcal conjugate vaccine
PHC: Population and housing census
RED: Reaching every district
SNNP: South Nation’s Nationalities and Peoples
SOS: Sustainable outreach service
USD: US dollar
VPD: Vaccine preventable disease
WHO: World Health Organization
